# Growth Method-Dependent and Defect Density-Oriented Structural, Optical, Conductive, and Physical Properties of Solution-Grown ZnO Nanostructures

**DOI:** 10.3390/nano7090266

**Published:** 2017-09-10

**Authors:** Abu ul Hassan Sarwar Rana, Ji Young Lee, Areej Shahid, Hyun-Seok Kim

**Affiliations:** Division of Electronics and Electrical Engineering, Dongguk University-Seoul, Seoul 04620, Korea; a.hassan.rana@gmail.com (A.u.H.S.R.); ljy010425@naver.com (J.Y.L.); areejshahid.146@gmail.com (A.S.)

**Keywords:** ZnO, defects, structural properties, convection, microwave, nanostructures, hydrothermal

## Abstract

It is time for industry to pay a serious heed to the application and quality-dependent research on the most important solution growth methods for ZnO, namely, aqueous chemical growth (ACG) and microwave-assisted growth (MAG) methods. This study proffers a critical analysis on how the defect density and formation behavior of ZnO nanostructures (ZNSs) are growth method-dependent. Both antithetical and facile methods are exploited to control the ZnO defect density and the growth mechanism. In this context, the growth of ZnO nanorods (ZNRs), nanoflowers, and nanotubes (ZNTs) are considered. The aforementioned growth methods directly stimulate the nanostructure crystal growth and, depending upon the defect density, ZNSs show different trends in structural, optical, etching, and conductive properties. The defect density of MAG ZNRs is the least because of an ample amount of thermal energy catered by high-power microwaves to the atoms to grow on appropriate crystallographic planes, which is not the case in faulty convective ACG ZNSs. Defect-centric etching of ZNRs into ZNTs is also probed and methodological constraints are proposed. ZNS optical properties are different in the visible region, which are quite peculiar, but outstanding for ZNRs. Hall effect measurements illustrate incongruent conductive trends in both samples.

## 1. Introduction

There is wide interest and research into oxide nanomaterials, including binary oxides, for example, ZnO, CuO, MgO, TiO_2_, and SnO_2_; ternary oxides, for example, BaTiO_3_, PbTiO_3_, BiFeO_3_, and KNbO_3_; and complex compounds, for example, Ba_1−*x*_Sr*_x_*TiO_3_, La_0.325_Pr_0.300_Ca_0.375_MnO_3_, and La_0.5_Ca_0.5_MnO_3_, due to their distinct geometries and cutting-edge physical and chemical properties [1]. ZnO has attracted significant attention because of its wide bandgap, and particular electrical, optical, structural, physical, chemical, piezo, and thermoelectric properties [2,3,4,5]. The occurrence of ZnO in polymorphic nanoscale shapes, such as nanorods (ZNRs), nanowires, nanoflowers (ZNFs), nanostars, nanoparticles, nanotubes (ZNTs), tetrapods, and polypods, further enhances its ambit under the wide canvas of myriad applications, such as field effect transistors, light-emitting diodes, ultraviolet lasers, photodetectors, thermo- and piezo-nanogenerators, solar cells, sensors, and so forth [6,7,8,9,10,11,12].

Optimized growth methods have occupied a foreground in the fabrication of ZnO nanostructures (ZNSs). The most eminent methods for ZNS synthesis are gas phase reaction, liquid phase deposition, metal organic chemical vapor deposition (MOCVD), vapor liquid solid, thermal evaporation, pulsed laser deposition, molecular beam epitaxy (MBE), thermal evaporation, and aqueous chemical growth (ACG) [13,14,15,16,17]. ACG, also called the hydrothermal method, is one of the most effective methods for ZNS growth due to simple setup, low cost, and green chemistry aspects [18]. However, the dilemma envisaged with the process are long heating times and temperature gradients in the solution autoclave, which can affect the structural and crystalline properties of ZNSs.

Microwave-assisted growth (MAG) methods have been recently proposed to address these issues [19]. MAG exploits the benefits of ACG, while addressing heat transfer problems and shortening fabrication time. Heat is produced by absorbance of longer wavelength and lower energy electromagnetic microwaves within the material rather than via convection. The advantages associated with MAG method are homogeneous heating profiles, higher heating rates, shorter fabrication time, precise control of the reaction mixture, selective heating with different microwave power, higher yields, and energy savings. Some disadvantages are high equipment cost; short penetration depth, which limits large-scale growth; arduous in situ monitoring; and very high homogeneous nucleation rates, which results in growth stoppage. However, the overall advantages outweigh the disadvantages, and MAG is usually recommended over the ACG method.

In this study, we present how to control the ZNSs crystal defect formation using convective ACG and irradiation MAG methods. Depending upon the crystal growth phenomenon, an in-depth comparison of growth, material, structural, optical, and conduction properties of ZNSs is performed. The experimental parameters, such as chemical constituents, molar concentrations of the precursor solutions, solution pH, ultimate growth temperature, and aspect ratio of the grown ZNSs were synchronized to allow fair comparison between ZNS properties grown with ACG and MAG methods. It was found that the ZnO morphology and defect density could be controlled by judiciously opting for the ZNS growth method. Furthermore, the structural, optical, etching, and conductive properties had a direct relation with ZNS defect density. The results are critically analyzed and all the antithetical trends have been propounded. 

## 2. Materials and Methods

### 2.1. Sample Preparation

Commercially available chemicals of analytical grade were purchased for the experiments. Aqueous solutions employed 18 MΩ de-ionized (DI) water. P-Si (100, 1–10 Ω∙cm) was used as a test substrate for ZNS growth. The P-Si substrates were cut into 1 × 1 inch segments and exposed to buffered oxide etchant to remove the insulating SiO_2_ layer at ambient conditions. After 2 min of immersion time, the substrates were cleaned with DI water and dried with N_2_ gas.

### 2.2. Thin Film Seeds

A buffer layer for ZNS growth is essential because of the large lattice mismatch between ZnO and Si (2.19 Å) [20]. Hence, a buffer layer of ZnO seeds was used to catalyze the ZnO growth species. Seeds were fabricated by mixing 0.022 M zinc acetate dihydrate [Zn(CH_3_COO)_2_·2H_2_O] (M_W_ 219.51 g/mol) in 10 mL n-propanol [C_3_H_8_O] (M_W_ 60.10 g/mol). The mixture was well stirred or sonicated for 30 min. The solution was ready to be deposited upon the substrate when the color changed from transparent to milky and back to transparent. The seed solution was then spin coated twice onto the substrate surface at 3000 RPM for 30 s. The spin coated samples were annealed at 110 °C for 2 min for the first coating, and at 300 °C for 60 min for the second coating to provide proper bondage between the seeds and substrate surface. 

### 2.3. ZnO Nanorods Using ACG and MAG Methods

ZNRs were grown using ACG and MAG methods. Separate growth solutions were made for the two methods: 50 mM zinc nitride hexahydrate [Zn(NO_3_)_2_·6H_2_O] (M_W_ 297.48 g/mol) was mixed with methenamine [C_6_H_12_N_4_] (M_W_ 140.186 g/mol) in DI water. Once the solutions were prepared, the seeded samples were immersed at the top of the solution to maximize heating effects within the solution. The solution bottles, with samples attached, were placed on a hot plate or in a 800 watt domestic microwave oven for the ACG and MAG methods, respectively. In ACG, a magnetic stirrer was placed at the base of the solution container, which was used to mobilize the reactants at the base, while the revolving disk in the microwave oven served the purpose in MAG method. The sensing probe of a wired thermometer was immersed in the solution autoclave to monitor the in situ temperature profile of the solutions. For synchronization, the ACG solution temperature was set above 100 °C to provide a perfect comparison between growth conditions and final products of both methods. After six hours, the ACG sample was removed from the solution, rinsed in DI water, and dried with N_2_ gas. For the MAG method, we used the solution replacement method to address growth stoppage. The solution was replaced four times after each 5-min microwave exposure. The sample was then rinsed in DI water and dried with N_2_.

### 2.4. ZnO Nanoflowers Using ACG and MAG Methods

ZNF growth was promoted using ammonium hydroxide [NH_4_OH] (M_W_ 35.05 g/mol) as a pH buffer, with 15–20 mL dissolved in 50 mM zinc nitride hexahydrate and methenamine solution, then stirred for 1 h to produce a homogeneous solution. The seeded substrates were immersed in the solutions under the ACG and MAG conditions described above. To ensure fair comparison between methods, the temperature and pH of the solutions were synchronized. The samples were removed after 6 h and 10 min for the ACG and MAG methods, respectively, rinsed in DI water, and dried with N_2_. 

### 2.5. ZnO Nanotubes Using ACG and MAG Methods

Various methods have been proposed for ZNT fabrication [21,22,23,24,25]. However, to allow fair comparison between ACG and MAG methods, we opted for ZNT growth using potassium chloride [KCl] (74.55 g/mol). This is one of the most effective, simplest, and safest metamorphosis methods, where formed ZNRs are etched into ZNTs. ACG and MAG ZNR samples were immersed in a 3–5 M KCl etching solution at 95 °C for 6 h. The samples were then removed, cleaned with DI water, and dried with N_2_.

### 2.6. Characterization Tools

To probe ZNS morphology, samples were imaged with scanning electron microscopy (SEM: Hitachi S-4800, Suwon, Gyeonggi-do, South Korea) operating at an emission energy of 25 KeV. Purity and crystalline quality were assessed via X-ray diffraction (XRD: Rigaku Ultima IV, Dongguk University, Seoul, South Korea) with Cu Kα radiation (λ = 0.15418 nm). The 2θ rage was taken from 20 to 50 degrees. Defect-centric optical properties were recorded with photoluminescence (PL: Accent RPM 2000, Suwon, Gyeonggi-do, South Korea) spectroscopy at scan rate of 15 pts/s with a laser excitation wavelength of 230–260 nm and power of 2.09 mW at room temperature and pressure (RTP). The PL range was set from 300 to 700 nm. The electrical characteristics and the carrier concentration were determined using Hall effect measurement system (ECOPIA AHT55T5, Dongguk University, Seoul, South Korea) at RTP in dark conditions. The in situ solution temperature and pH were monitored with a wired digital pH and thermometer. A Samsung domestic 850 watt microwave oven (Dongguk University, Seoul, South Korea) was used for MAG method. The photolithographic patterns were defined on the substrate with the help of Karl Suss MA 6 mask aligner (Dongguk University, Seoul, South Korea), and the metal contacts were made with E-beam metal evaporator (Dongguk University, Seoul, South Korea).

## 3. Results and Discussion

### 3.1. Growth Mechanism and Internal Chemistry

ZNS growth depends upon growth temperature to facilitate the chemical reactions,

$$C_{6}H_{12}N_{4}\;+\;6H_{2}O\;\overset{\mathrm{Heat}}{\to }4{\mathrm{NH}}_{3}\;+\;6\mathrm{HCHO},$$

$${\mathrm{NH}}_{3}\;+\;H_{2}O\;\overset{\mathrm{Heat}}{\to }{{\mathrm{NH}}_{4}}^{+}\;+\;{\mathrm{OH}}^{-},$$

$$\mathrm{Zn}{\left({\mathrm{NO}}_{3}\right)}_{2}\cdot 6H_{2}O\;\overset{\mathrm{Heat}}{\to }{\mathrm{Zn}}^{2+}\;+\;2{{\mathrm{NO}}_{3}}^{-},$$

and

$${\mathrm{Zn}}^{2+}\;+\;2{\mathrm{OH}}^{-}\;\overset{\mathrm{Heat}}{\to }\mathrm{Zn}{\left(\mathrm{OH}\right)}_{2}\;\overset{\mathrm{Heat}}{\to }\;\mathrm{ZnO}\;+\;H_{2}O,$$

where the reaction rates depend strongly on the heat transfer method [18,19].

**Figure 1:**
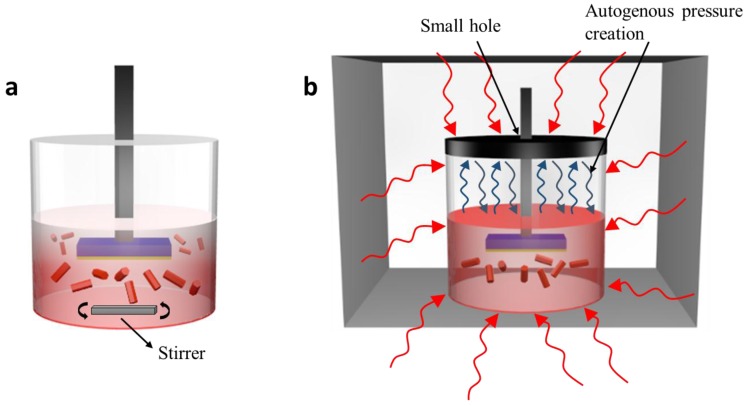
Heating profile for (**a**) aqueous chemical growth (ACG) and (**b**) microwave-assisted growth (MAG) methods.

Figure 1 demonstrates ZNS growth for ACG and MAG methods, with solution temperature shown by darker (hotter) and paler (cooler) red. The samples were studiously attached at the topmost possible position to scrutinize the distribution of heat inside the solution. Convective heating (ACG method, Figure 1a) produces a temperature gradient between the bottom and top of the solution. Black body radiation is convectively conducted into the solution, facilitating the reaction, and the reaction vessel only intercedes between energy transfer from the hotplate into the solution. The temperature gradient results in higher and lower reaction rates near the bottom and top surfaces, respectively, which leads to inhomogeneous ZNS growth. The stirrer at the base mobilizes the homogeneously nucleated large ZNRs at the bottom and the small ZNRs at the top surface, which are nucleated on the seeded substrate. A thin layer of ZnO seeds, with crystal orientation towards 0001, lowers the surface energy at the ZnO-Si interface and improves heterogeneous nucleation of the crystal nuclei on the seeded substrate rather than homogeneous nucleation in the solution [26]. The resulted orientation of the formed ZNRs is also towards the 0001 direction. Furthermore, temperature rise is also relatively slow, taking approximately 50 min for the solution to reach the required nucleation temperature. It is well established that ZnO crystal quality is growth temperature-dependent [27]. ZnO growth requires a particular amount of thermal energy for the derivation of chemical reactions and crystal growth. The prevalent temperature gradients in ACG provide bases for the difference in thermal energies provided to the reactants at different catalytic temperatures [28]. The said phenomenon affects the crystalline quality of the formed nanostructures.

We also believe that the said difference in the ACG ZNR dimensions is because of the formation, growth, and implosion of sono-chemical acoustic cavitations in the solution [29]. The stirrer at the base provides sound waves and shear kinetic energy to the reaction solution. The impinging sound waves, with a wavelength longer than the bond length of the reactants, do not have the ability to affect the formation energies of the reactants and cannot influence the chemical reaction directly [30]. However, the stirrer forms acoustic cavitations in the form of bubbles, which act as packets of high energy, temperature, and pressures inside the solution [31,32]. The revolving packets act as carriers of very high energy, which is converted into heat upon implosion and speeds up the chemical activity in the vicinity, while the reaction rate remains the same elsewhere. The phenomenon results in the formation and nucleation of disproportionate dimension ZNSs in the solution.

In contrast, the MAG heating profile is quite smooth and enticing, as shown in Figure 1b. The 2.85 GHz microwaves bombard the reaction flask, and hence the solution, from all sides. The solution is heated by ionic conduction of the dissolved chemicals and dipolar polarization of water molecules. Microwaves are relatively evenly distributed within the solution, and the solution experiences a steep and homogeneous temperature rise in approximately 2 min, which stimulates both nucleation and crystal growth [33]. It is noteworthy that the ionic conduction has more profound heat generating capacity than dipolar polarization, which has considerable implications on nanomaterial growth in ionic liquids. Furthermore, operational parameters, such as reaction temperature, irradiation power, and vessel pressure, must be precisely controlled to ensure a smooth microwave interaction with the material. Hence, a specially designed reaction container that facilitates autogenous pressure creation within the reaction chamber expedites the chemical reactions.

### 3.2. Defect Density and Conductive Properties of ZNRs Grown with ACG and MAG Methods

ZnO is naturally an n-type semiconductor, but the longstanding controversy to discern the unintentional n-type ZNS characteristics is yet a moot point in research and development. Non-stoichiometry during ZNS growth is cited as the main reason for this dilemma, and is the origin of the prevalent stalemate. Intrinsic defects within the ZnO crystal structure are pivotal for feasible theories of the origin of n-type characteristics. Oxygen vacancies (V_o_) and zinc interstitials (Zn_i_) are considered as potential donors in some literature, with hydrogen (H) also sometimes considered important [34,35,36]. Mollwo, Thomas, and Lander first proposed the theory of H donor in intrinsic ZnO, which was later substantiated by Van de Walle [37,38]. It is believed that H replaces V_o_ via four-fold coordination with neighboring atoms in ZnO crystal structure, and acts as a donor. Recently, many groups have countered the concept of H being an intrinsic donor impurity with experimentation and hypotheses [39]. Despite the enduring controversy, the V_o_ concept is central to all the proposed theories on the origin of unintentional n-type conductivity in intrinsic ZnO.

Table 1 shows the measured electrical characteristics; carrier concentration, resistivity, and mobility; of samples grown with ACG and MAG methods. The characterization was done with the Hall effect measurement system. The ZNRs were grown on an insulating glass substrate and ohmic indium contacts were fabricated on the four corners of the samples to provide optimal results. The In dots were fabricated directly on ZNRs by defining a four-spot window via shadow mask photolithography and deposition via e-beam metal evaporation. We did not use any top layer for the deposition of metal contacts and the contacts were made directly upon the ZNRs. The four probes were attached on the contacts with alternating rotations for optimization. Both samples show n-type conductivity, but carrier concentration in ACG samples is very high compared to MAG samples because of the plethora of donor defects. The high-power microwave irradiations provide sufficient thermal energy to the atoms to nucleate on the proper crystal lattice points, reducing the probability of V_o_ production. Although, the crystalline quality of MAG samples is superior, a steep descent in carrier concentration is the reason for high resistivity. On the contrary, the inefficient convective heating profile of ACG provides a platform for the production of V_o_ in the crystal lattice and hence enhanced n-type conductivity. The following equations were used to govern the relation between Hall voltage, carrier concentration, and mobility of the samples. The relation for Hall voltage (V_H_) is:

(1) $$V_{H}=\frac{B_{z}I_{x}}{ned}$$

Hence, the majority carrier concentration (n) would be:

(2) $$n=\frac{B_{z}I_{x}}{V_{H}ed}$$

where *B_z_* is the magnetic field towards z-direction, *I_x_* is drift current, *d* is film thickness, and *e* is electronic charge magnitude. The relation between majority carrier concentration (n) and mobility (${\text{µ}}_{n}$) of the samples was governed by:

(3) $${\text{µ}}_{n}=\frac{LI_{x}}{enV_{x}Wd},$$

where *L* is the length and *W* is the width of the sample under test, and *V_x_* is the drift voltage. It is evident from (3) that the mobility of the samples is inversely proportional to the majority carrier concentration, which is the primary reason of very high mobility in MAG ZNRs as compared to ACG ZNRs, as seen in Table 1. Hence, it is proved that ZnO carrier concentration is growth method-dependent and can also be controlled by an optimal use of a growth method rather than doping. The conductive properties of both samples are in accordance with XRD and PL data. Furthermore, the defect density influenced the ZnO structural, optical, and etching behaviors, as shown in the next.

### 3.3. Methodological Constraints for Nanorod Growth Via ACG and MAG Methods

**Figure 2:**
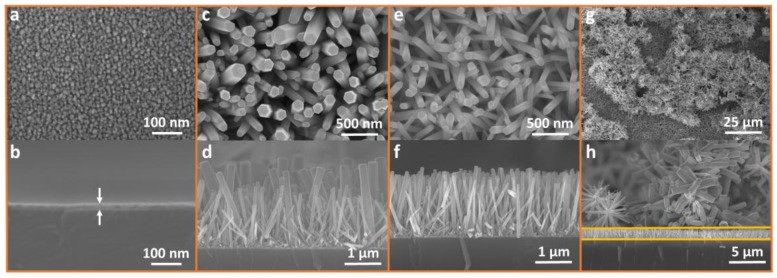
Scanning electron microscope (SEM) (top and side views, respectively): (**a**,**b**) Seed; (**c**,**d**) ZnO nanorods (ZNRs) fabricated using the ACG method; (**e**,**f**) ZNRs fabricated using the MAG method; (**g**,**h**) ZnO debris on the surface of vertical ZNRs.

Figure 2 shows SEM images of ZNRs grown with ACG and MAG methods. Figure 2a,b shows top and the cross-sectional SEM images of the buffer layer of ZnO seeds, respectively. Seeds are necessary to provide the nucleation energy required for ZNR growth, lower interfacial energy at the ZnO substrate interface, and minimize the large lattice mismatch and ultimately the stress and the strain between both materials. The 0.022 M zinc acetate seeds were spin coated twice upon the surface to provide a 15-nm seed layer. Double seed coating was chosen to reduce fabrication time.

Figure 2c,d shows top and cross-sectional images, respectively, of ZNRs grown with the ACG method at temperatures above 100 °C. The resultant ZNRs are hexagonal and densely populated. However, ZNR dimensions are eccentric and inhomogeneous, due to inept convective heating process and stirring. In contrast, ZNRs grown by the MAG method are also densely populated, but relatively homogeneous and immutable, as shown in Figure 2e,f. To allow fair comparison, the MAG ZNR lengths were synchronized with ACG ZNR lengths to approximately 2 μm. In this regard, the fabrication of ZNRs via a facile MAG solution-replacement method is an ideal method to adopt because it is easy to control the length of ZNRs within the solution [19]. Using 25 mM solution, ZNR length increased 250 nm per solution-replacement cycle on average, which doubled to 500 nm per solution-replacement cycle for a molar concentration 50 mM. Hence, replacing the solution four times produced 2 μm ZNRs. Above all, the ZNRs were grown in an amazingly short fabrication time of 20 min, in contrast to 6 h using the ACG method.

Another hitch associated with the ACG method is deposition of debris ZNSs on the surface of the active ZNR layer. Some ZNRs were homogeneously nucleated inside the solution, mobilized by the stirrer, and deposited on the substrate surface. Longer ACG fabrication time exacerbates debris deposition. As shown in Figure 2g, the debris is quite dense and the active area is covered with debris. The cross-sectional view of Figure 2h shows the havoc played by the debris, where the debris layer thickness is quite high compared to the 2-μm vertical ZNRs enclosed in the yellow strip. The debris could be proved devastating and offers an utter mess to measure the material, optical, and electrical properties of vertically aligned ZNRs, and requires multiple measurements to acquire meaningful outcomes for useful ZNRs. Because of very short fabrication time, such debris was not deposited in ZNRs grown with MAG method, as shown in the background of Figure 2f. A short-term solution to address debris is to clean the sample with de-ionized (DI) water immediately after removing it from the growth solution. This removes much of the debris from the surface, since it is poorly attached compared to the desired vertical ZNRs.

### 3.4. Methodological Constraints for Nanoflower Growth via ACG and MAG Methods

**Figure 3:**
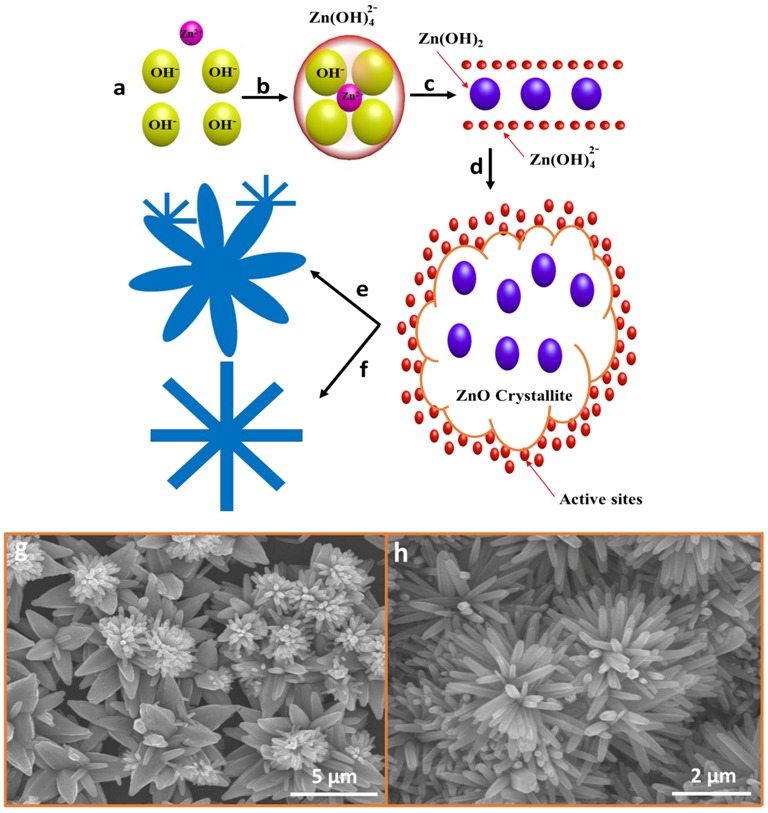
(**a**–**f**) Process flow for ZnO nanoflowers (ZNFs) growth using the ACG and MAG methods, (**g**) SEM image of ZNF using the ACG method, and (**h**) SEM image of ZNF using the MAG method.

ZNFs were fabricated via NH_4_OH treatment, one of the most effective growth methods [19]. The name of the structure reflects the geometrical similarity to a flower. Most research groups use NaOH for ZNF growth, but we find NH_4_OH treatment more beneficial and efficient. The detailed growth mechanism is portrayed in Figure 3. Before the addition of NH_4_OH, methanemine is decomposed to provide OH^−^ ions, which then react to form Zn(OH)_4_^2−^ growth units and Zn(OH)_2_ nuclei. However, at low pH, the nuclei are more than the growth units, which halts ZNF growth, and only ZNRs are formed. With the addition of NH_4_OH as a mineralizer and pH buffer, OH^−^ ion population is significantly increased in the solution. The initial solution pH was approximately 6.8, and increased to as much as 12 after NH_4_OH addition. Zn^2+^ and OH^−^ ions react to form ZnO nuclei and growth units, as shown in Figure 3a,b. When the pH was elevated to 12, the growth units were more than ZnO nuclei and aligned via electrostatic attractive forces between the ZNF nuclei, as shown in Figure 3c. The growth units and nuclei start amalgamating through Ostwald ripening, consuming smaller crystals into larger crystals. Due to very low surface energy under the influence of homogeneous nucleation, a number of ZnO nuclei amalgamate to form ZnO crystallites, as shown in Figure 3d. All the active sites circumambulate at the outer edges of the crystallite to provide a base for petals, and are ready to be grown into ZNFs at high temperatures. The process flow for both ACG and MAG methods remain the same until this point. 

Figure 3e,g shows the schematic for a single ZNF and SEM image, respectively, grown with the ACG method. As discussed above, the homogeneous nucleation rate is higher and lower near bottom and top surfaces of the reaction flask, respectively, which leads to inhomogeneous ZNF growth. Thus, the SEM image shows that ZNFs are quite large, but small ZNFs also start nucleating at the petals and at different centers of the already grown ZNFs. Relatively large ZNFs are formed at the bottom of the flask, which are mobilized by the stirrer and adsorbed at the substrate surface at the top edge of the solution flask. Simultaneously, small ZNFs are homogeneously nucleated and adsorbed upon the petals of already formed ZNFs, which produces inhomogeneous ZNF growth for the ACG method. Another point to ponder is the difference in the diameter of the top (0001) and bottom (000ī) surfaces of the ZNF petals. The underlying reason for this phenomena is the difference in growth rate of various ZNF planes: V(0001) > V(ī0īī) > V(ī010) > V(ī011) > V(000ī) [40]. The bottom 000ī surface, with the lowest growth rate, becomes stable, and the top 0001, with the highest growth rate, erodes at the growth stoppage point because of the presence of the sample inside the solution for such a long time at high temperatures in the ACG method. Hence, pointed-tip ZNFs are formed (see Figure 3e,g). 

In contrast, ZNF growth via the MAG method is relatively homogeneous and quick, as shown in the schematic and SEM image of Figure 3f,h, respectively. All the flower petals have a similar length and diameter, and the ZNFs are quite dense and homogeneously distributed across the substrate surface. The homogeneous growth is because of MAG efficient heat transfer. Erosion at the top 0001 surface is not evident, because of the very short fabrication time: the samples were ready to be removed from the solution within just 10 min for the MAG method. It is evident that the erosion would be seen at the top 0001 surface in MAG if the sample is left under MAG growth conditions beyond 10 min. However, that is not required because the precursor solution has already been saturated by MAG in 10 min.

### 3.5. Defect-Centric Etching of Nanorods into Nanotubes

ZNTs have paramount importance because of their distinct chemical properties, hollow structure, high surface-to-volume ratio, high anisotropy, and current carrying capability. However, ZNT growth is quite challenging. We propose ZNT growth via defect-centric etching of ZNRs grown with the ACG and MAG methods. Defect-centric etching is the only known feasible method for ZNT formation. Hence, it is important to compare the practical application of this method on ZNRs grown with the ACG and MAG methods [41].

**Figure 4:**
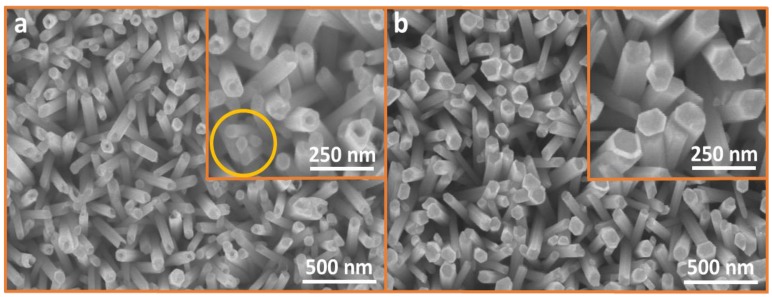

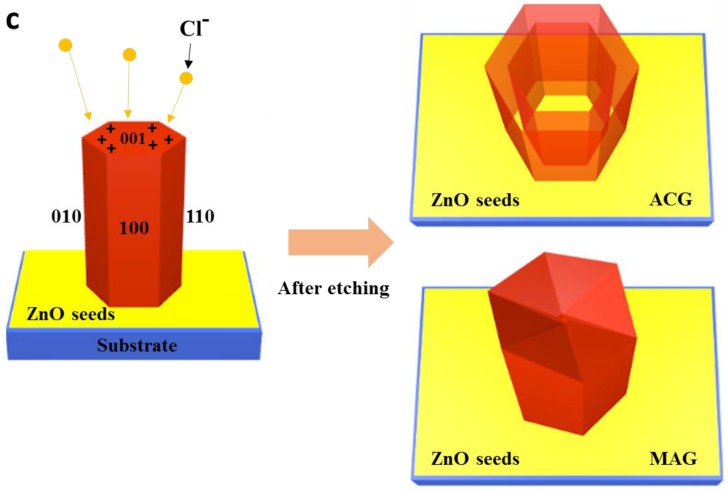
SEM images of ZnO nanotubes (ZNTs) (inset: magnified image), formed via defect-centric etching of ZNRs grown with (**a**) ACG and (**b**) MAG methods; (**c**) Etching mechanism for ACG and MAG ZNRs.

Figure 4a shows the SEM image of ZNTs formed via defect-centric etching of ZNRs grown with the ACG method in a KCl solution. The inset shows a corresponding higher magnification portion. Almost all the ZNRs are etched at the core towards the bottom, and a hollow tubular structure is formed. The tube walls are quite thick and the hole is formed only at the core. The tube shape remains hexagonal after etching, which means the etching was only performed in the unstable ZNR core. Hence, it is inferred that the formation of ZNTs from ZNRs grown with the ACG method is feasible because of the polycrystalline structure and the profusion of defects created within the ZNR crystal structure during the growth process. The absence of etching for a few of the inhomogeneous, smaller-diameter ZNRs are highlighted in the inset of Figure 4a. Thus, etching does not only depend upon defect density, but also diameter and area available for etching. Etching appears to be proportional to defect density and ZNR diameter. Trimming of such a small diameter ZNRs into ZNTs, which is quite arduous, has not been propounded previously, and is successfully performed here for the first time.

Figure 4b shows the SEM for MAG method ZNRs etched into ZNTs in a KCl solution with the corresponding high magnification image in the inset. None or only very few ZNRs are etched, but the etching stops at the surface. Furthermore, in the case of partial etching, it is found that the core is etched with the most stable ZNR lateral walls. The absence of defect-centric etching is because of the pure crystalline structure of ZNRs grown with the MAG method. Because of the immaculate nature of the MAG method, only a few defects are incorporated in the crystalline structure, which makes it difficult for the etchant to trim the formed ZNRs into hollow ZNTs. Furthermore, the few existing defects tend to be at the ZNR surface, which results in a skimpy etching profile.

Figure 4c further clarifies how the Cl^−^ ions are adsorbed on the polar surface of the formed ZNRs and etch the defect-rich ACG ZNRs from core to base. A hollow surface towards the bottom is seen with quite thick and stable lateral walls. This also shows how MAG ZNR was either not etched or etched only at the surface with the dissolution of most stable lateral walls. These visualizations are in accordance with X-ray diffraction (XRD), photoluminescence (PL), and Hall effect measurements of the samples.

### 3.6. XRD for ZNRs Grown with ACG and MAG Methods

**Figure 5:**
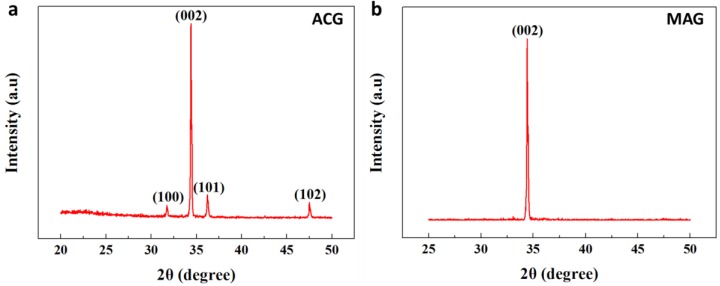
ZNR X-ray diffraction (XRD) profiles: (**a**) the ACG and (**b**) the MAG method.

The structural and crystalline properties of ZNRs grown with ACG and MAG methods were investigated using XRD, as shown in Figure 5. The multiple peaks across 100, 002, 101, and 102 in Figure 5a and a single peak across 002 in Figure 5b correspond to the hexagonal phases in ZNRs, confirming the wurtzite nature of both samples. The incorporation of multiple peaks in ACG ZNRs imply the deterioration of ZnO 002 texture. However, the highest peak in both samples were across 002, which confirms ZNR vertical alignment along the *c*-axis perpendicular to the substrate, in accordance with the SEM data. The multiple peaks and a single peak also show that ACG and MAG ZNRs are poly and single crystalline, respectively. 

Stress and the strain in the crystal structures depend upon multiple factors, such as the lattice mismatch, 2θ position of the 002 peak, and the lattice constant (*C*_o_) of the grown crystals. Extrinsic stress/strain was not considered because both samples were grown on p-Si with a ZnO buffer layer, but an in-depth structural analysis has been performed for intrinsic stress/strain levels depending upon the growth method used. The lattice constants, a and c, of ZnO were calculated via Braggs law:

(4) 2dsinθ=nλ

where *d* is the spacing between the lattice planes of Miller indices, *n* is order of diffraction which is normally taken as 1, *λ* is X-ray wavelength (1.54 Å), and *θ* implies Bragg’s angle. The lattice constant for (100) plane is calculated by the relation:

(5) $$a=\frac{\lambda }{2sin\theta }\sqrt{\frac{4}{3}\left(h^{2}+hk+k^{2}\right)+{\left(\frac{a}{c}\right)}^{2}l^{2}}$$

(6) $$a=\frac{\lambda }{\sqrt{3}sin\theta }$$

where *θ* is the diffraction angle responding 100 peak. The lattice constant *c* for 002 plane is calculated by the relation:

(7) $$c=\frac{\lambda }{2sin\theta }\sqrt{\frac{4}{3}{\left(\frac{c}{a}\right)}^{2}\left(h^{2}+hk+k^{2}\right)+l^{2}}$$

(8) $$c=\frac{\lambda }{sin\theta }$$

where *θ* corresponds to 002 peak. The 2θ value of the 002 peak and *C_o_* for stress-free bulk ZnO are 34.42 and 0.5205 nm, respectively [42]. The 2θ of samples grown with the ACG and MAG methods were 34.31 and 34.43, respectively. The 0.1 degree left-shift in ACG samples was caused by stress [43]. The 2θ value of MAG samples are more near to the bulk ZnO, which is the direct result of intrinsic stress/strain relaxation during MAG process. The process minimizes the surface energy of the film and adds value to the crystallinity of the sample. Furthermore, the lattice constant *C_ACG_* is 0.5238 and *C_MAG_* is 0.5201 nm. ACG samples show tensile stress (*C_ACG_* > *C*_o_), whereas the MAG samples show compressive stress (*C_MAG_ < C_o_*), so the strain along the *c*-axis, $\left[\left(C-C_{o}\right)/C_{o}\right]\times 100$, was 63% (compressive) and 7% (tensile) for ACG and MAG samples, respectively. Thus, the stress and strain levels in MAG samples are almost negligible, which validates the methodological efficacy of the MAG method.

The full width at half maximum (FWHM) of the 002 peak and grain size (D) are indicative of the crystalline quality of the ZNRs. The Scherrer formula was used to calculate D [44,45],

(9) D=(0.89λ)/(B cosθ),

where *λ* is the X-ray wavelength (0.15418 nm), *B* is the FWHM in radians, and *θ* is the diffraction angle. Using the FWHM shown in Figure 5a,b, D = 27 nm and 68 nm, for ACG and MAG samples, respectively. D is indicative of crystal quality and yield stress in the structure. Larger D implies that less driving force is required to move a dislocation pileup near the edges from one crystallite to another. MAG process stimulates the grain boundary migration, which results in facile coalescence of crystallites and ponder large grain growth. In short, unlike ACG, MAG provides sufficient energy to the atoms to expeditiously occupy the legitimate crystal sites on the crystal lattice. Hence, material with smaller grains (ACG sample) exhibits higher yield stress than material with larger grains (MAG sample). Yield stress can also be governed by the Hall-Petch equation,

(10) $$\sigma y=\sigma_{0}+\frac{ky}{\sqrt{d}},$$

where σ_y_ is the yield stress; *σ_o_* is the material constant; *k_y_* is the strength coefficient specific to each material; and *d* is the average grain diameter. Grain diameter is inversely proportional to the yield stress, which implies that ACG samples will have more yield stress than MAG samples. Hence, it is proved that stress/strain in the films are growth process-dependent rather than of thermal origin. Moreover, the crystalline quality of MAG samples is comparable to samples grown with expensive and sophisticated equipment, such as MOCVD and MBE [46,47]. The bond length (L) of ZnO nanostructures grown with ACG and MAG methods were calculated via:

(11) $$L=\sqrt{\frac{a^{2}}{3}+{\left(\frac{1}{2}-u\right)}^{2}c^{2}}$$

where *u* parameter, which depends upon *a*/*c* ratio in wurtzite structures, can be calculated as:

(12) $$u=\frac{a^{2}}{3c^{2}}+0.25$$

The calculated values of L, along with other structural parameters, are given in Table 2.

### 3.7. ZNR Optical Properties from ACG and MAG Methods

**Figure 6:**
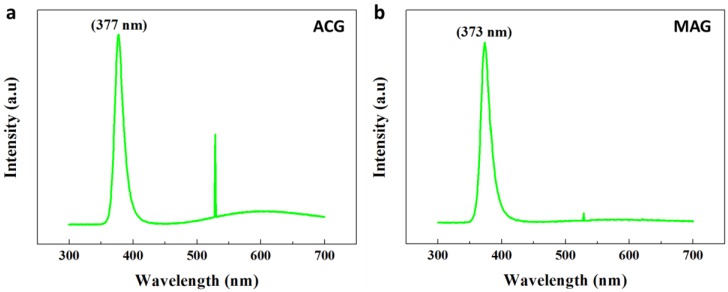
ZNR Photoluminescence spectra: (**a**) the ACG and (**b**) the MAG method.

ZNR absorption and emission spectroscopy are important research and development parameters due to the unique optical properties of ZnO. The optical properties were measured with PL Accent RPM 2000 at room temperature. The nanostructure luminescent properties were strongly dependent upon the growth method used and the nanostructure crystal growth. Figure 6a shows the PL response of ACG ZNRs, displaying an orthodox high-intensity UV peak at 377 nm (3.27 eV) and a broad peak in the visible range. The high-intensity UV peak, with FWHM ~16.5 nm, is caused by free exciton recombination, and the broad visible emission is due to defects formed within the crystal during fabrication [48]. Various contradictory theories have been proposed, including V_o_, oxygen interstitials, zinc vacancies, and zinc interstitials Zn_i_, for the luminescent recombination centers. However, the presence of V_o_ is the most pertinent theory for the broad luminescence band in the visible region. The most plausible charge states for V_o_ in the crystal are neutral (V°_o_), singly ionized (V^+^_o_), and doubly ionized (V^2+^_o_) [49]. Singly ionized vacancies (V^+^_o_) are the most susceptible to act as a green emission and electron recombination centers [50]. A peculiar large and sharp kink at 528 nm was also observed in the visible region.

Similarly, Figure 6b shows PL spectra for MAG ZNRs, which is quite distinct from ACG ZNRs. The UV peak was 373 nm (3.30 eV) with high-intensity and low FWHM (~15 nm), displaying significantly better optical quality compared to ACG ZNRs. Another important criterion to judge optical performance is the ultraviolet-to-visible emission ratio, and MAG ZNRs have ~3 times the value of ACG ZNRs, due to their higher intensity UV peak and very low peak intensity in the visible region [28]. MAG ZNRs also show a flat band in the visible region because of the absence of defect-centric recombination centers in the crystal structure. The authenticity of the results could be checked by matching the structural and optical performance of the same ZNRs [51]. It is found that the optical performance of the samples, such as the defect density, crystalline quality, and optical structure of ZNRs, is in accordance with the findings of the structural properties of the same ZNRs in Figure 5.

Another point to ponder in the PL data is the deviation of ACG ZNR UV peak from 373 nm in MAG ZNRs to 377 nm. We believe that the observed red shift and the reduction in near band edge emission in ACG ZNRs are because of band gap renormalization (BGR) effect. It has already been established that the free electron density of ACG ZNRs is far superior to MAG ZNRs (Table 1). This high electron density results in BGR via many body effect and free carrier screening inside the structure and is the primary reason for red shift in the sample [52]. Furthermore, slightly large diameter and high lattice stress in ACG ZNRs could be secondary reasons for this red shift [53]. Additionally, the sharp peaks at 528–530 nm in both ACG and MAG ZNRs are quite unusual for ZnO. Previously, we believed that the sharp peaks were because of the presence of confined defects (V_o_) in ZnO lattice. However, the sharp peaks are actually the PL laser response imbibed during the data acquisition with PL: Accent RPM 2000.

## 4. Conclusions

We present method-dependent and crystallization-oriented growth, material, structural, optical, and conductive properties of ZNSs grown using the classic ACG and emerging MAG methods. The effects of convective (ACG) and radiative (MAG) processes were discussed in the context of ZNS crystal growth. The two methods could be exploited to control the ZnO defect density. The ACG ZNRs and ZNFs showed inhomogeneous growth because of convective temperature gradients, and sono-chemical acoustic cavitations in the solution and MAG ZNRs led to homogeneous growth trends because of immutable irradiative heating. ACG and MAG ZNSs were poly and single crystalline, respectively, which provided further verification of the optical and electrical properties. Conversion of MAG ZNRs to ZNTs was quite difficult because of their defect-free structure. Furthermore, MAG ZNRs showed superior optical profile and flat PL response in the visible region. Crystalline and growth properties provided the explanation for high and low n-type intrinsic conductivity in ACG and MAG samples, respectively. Further research on growth and methodological constraints of ACG and MAG methods is required to address basic problems for ZNS growth, and illuminate fresh opportunities for application-oriented experimental and theoretical studies on ZNS growth via ACG and MAG methods.

## Figures and Tables

**Table 1:** Electrical and conductive properties of samples grown with ACG and MAG methods.

Growth Method	Carrier Concentration (cm^−3^)	Resistivity (Ω∙cm)	Mobility (cm^2^/V-s)
ACG	1.8 × 10^17^	31.8	1.05
MAG	1.08 × 10^14^	1010	56.8

**Table 2:** Structural parameters of samples grown with ACG and MAG growth methods. Full width at half maximum (FWHM); grain size (D); bond length (L).

Growth Method	*c* (Å)	2θ (Degree)	FWHM (Degree)	D (nm)	L (Å)	Strain (%)
ACG	5.238	34.38	0.311	26.86	1.9751	63
MAG	5.201	34.43	0.122	68	1.9769	07
